# Effects of Photoperiod on Survival, Growth, Physiological, and Biochemical Indices of Redclaw Crayfish (*Cherax quadricarinatus*) Juveniles

**DOI:** 10.3390/ani14030411

**Published:** 2024-01-26

**Authors:** Xiangxing Nie, Cuixue Huang, Jie Wei, Yakun Wang, Kunhao Hong, Xidong Mu, Chao Liu, Zhangjie Chu, Xinping Zhu, Lingyun Yu

**Affiliations:** 1 School of Fishery, Zhejiang Ocean University, Zhoushan 316000, China; niexiangxing@zjou.edu.cn (X.N.); huangcuixue@zjou.edu.cn (C.H.); czj0501@zjou.edu.cn (Z.C.); 2 Key Laboratory of Tropical and Subtropical Fishery Resources Application and Cultivation, Ministry of Agriculture and Rural Affairs, Pearl River Fisheries Research Institute, Chinese Academy of Fishery Sciences, Guangzhou 510380, China; zjweijie@prfri.ac.cn (J.W.); wangyk@prfri.ac.cn (Y.W.); hongkunhao@prfri.ac.cn (K.H.); zhuxinping_1964@163.com (X.Z.); 3 Key Laboratory of Prevention and Control for Aquatic Invasive Alien Species, Ministry of Agriculture and Rural Affairs, Guangdong Modern Recreational Fisheries Engineering Technology Center, Pearl River Fisheries Research Institute, Chinese Academy of Fishery Sciences, Guangzhou 510380, China; muxd@prfri.ac.cn (X.M.); liuchao@prfri.ac.cn (C.L.)

**Keywords:** aquaculture, circulating water culture, environmental factor, body color, gene expression

## Abstract

**Simple Summary:**

In aquaculture, the survival and health of aquatic organisms are influenced greatly by external environmental factors. Photoperiod is one of the key environmental factors that affect the early growth, survival, metabolism, and immune system of crustaceans significantly. The aim of the present study was to investigate the impact of photoperiod on the growth, survival, enzyme activity, body color, and expression of growth-related genes in juvenile redclaw crayfish. Five photoperiod experimental groups were established (0L:24D, 6L:18D, 12L:12D, 18L:6D, and 24L:0D), each with three replicates, and reared for 30 days. The group with an 18L:6D photoperiod had higher juvenile crayfish survival rates, better growth performance, stronger antioxidant stress response, and immune defense capabilities. Additionally, artificial photoperiods altered the body color of the redclaw crayfish. The results of the present study lay a foundation for the elucidation of the mechanisms underlying photoperiod regulation of the physiological and biochemical responses of juvenile redclaw crayfish.

**Abstract:**

Through a 30-day experiment, this study investigated the effects of five photoperiods (0L:24D, 6L:18D, 12L:12D, 18L:6D, and 24L:0D) on the survival, enzyme activity, body color, and growth-related gene expression of redclaw crayfish (*Cherax quadricarinatus*) juveniles. The results showed that *C. quadricarinatus* juveniles under 18L:6D and 24L:0D photoperiods exhibited the highest survival rate, which was significantly higher than the survival rates of juveniles under the other three photoperiods (*p* < 0.05). However, the 0L:24D group had the highest final body weight and weight gain rate, significantly surpassing those of the 12L:12D, 18L:6D, and 24L:0D groups (*p* < 0.05). Regarding enzyme activity and hormone levels, juveniles under the 18L:6D photoperiod exhibited relatively higher activity of superoxide dismutase (SOD), acid phosphatase (ACP), and lysozyme (LZM) enzymes than those under other photoperiods, but their levels of melatonin and cortisol were relatively low. In addition, the 24L:0D group showed the highest malondialdehyde (MDA) content. Analysis of gene expression levels revealed that retinoid X receptor (*RXR*) and α-amylase (*α-AMY*) genes in *C. quadricarinatus* juveniles exhibited significantly higher expression levels under the 18L:6D photoperiod than those under the other four photoperiods (*p* < 0.05). With increasing daylight exposure, the body color of *C. quadricarinatus* changed from pale blue to yellow–brown. In summary, *C. quadricarinatus* juveniles achieved high survival rates, good growth performance, strong antioxidant stress response, and immune defense capabilities under an 18 h photoperiod. Therefore, in the industrial seedling cultivation of redclaw crayfish, it is recommended to provide 18 h of daily light. Further, the study demonstrated the ability to manipulate the body color of *C. quadricarinatus* through controlled artificial photoperiods. These findings provide essential technical parameters needed for the industrial cultivation of *C. quadricarinatus* juveniles.

## 1. Introduction

Several environmental factors, including temperature [1], salinity [2], light exposure [3], ammonia nitrogen [4], and nitrite [5], influence the growth, survival, development, reproduction, distribution, and behavior of aquatic animals in the natural environment. Among these factors, light exposure emerges as a fundamental external influence in ecosystems, manifesting primarily through three distinct dimensions: light intensity, spectrum, and photoperiod. Organisms have developed an internal circadian rhythm system to adapt to continuously changing light conditions resulting from Earth’s rotation and revolution [6]. Of these, photoperiod is considered the most important condition influencing the circadian rhythm of animals [7]. For instance, the entire life cycle of most teleost fish is influenced by light [8]. Under a 14 h photoperiod, Senegal sole (*Solea senegalensis*) showed relatively high survival rates [9]. Conversely, the survival rate of Atlantic cod (*Gadus morhua*) decreases under continuous illumination [10]. Photoperiod has been shown to affect processes like molting [11], competitive behavior [12], and feeding and growth [13] in lower-ranked crustaceans. However, there remains a scope for further comprehensive exploration of the physiological and biochemical regulatory mechanisms of photoperiod in crustaceans.

The response of animals to changes in external light conditions involves complex physiological and biochemical processes, and various biochemical substances [14,15,16]. Among these substances, melatonin (N-acetyl-5-methoxy-tryptamine) plays a vital regulatory role in the circadian rhythms of animals [17]. Studies on crustaceans have shown that melatonin level is influenced by changes in photoperiod. The rhythmic secretion of melatonin by Chinese mitten crab (*Eriocheir sinensis*) and the crab (*Neohelice granulate*) can be disrupted under constant light conditions [15,18]. Furthermore, as a stressor, drastic changes in photoperiod can cause crustaceans to produce a series of responses, which cause variations in reactive oxygen species (ROS) levels and lead to oxidative stress and negative impacts [16,19]. Cortisol is an essential indicator of internal environmental stability in crustaceans. An increase in cortisol level is typically considered a sign of stress [14]. Studies on crustaceans have revealed that changes in photoperiod induce stress in crustaceans. For example, mud crab (*Scylla paramamosain*) juveniles under daily 18 h light conditions have a higher cortisol level and the lowest survival rate compared to other photoperiod groups [20]. Moreover, changes in photoperiod could lead to variations in the secretion levels of antioxidant enzymes and immune factors among crustaceans [16,19]. For example, the superoxide dismutase (SOD) activity of giant freshwater prawn (*Macrobrachium rosenbergii*) decreases with a decrease in daylight exposure, whereas lysozyme (LZM) activity increases with an increase in daylight exposure [21]. Acid phosphatase (ACP) activity in pacific white shrimp (*Penaeus vannamei*) increases with an increase in daylight exposure, whereas malondialdehyde (MDA) content increases with an increase in daylight exposure [16]. Interestingly, these changes in photoperiod also induce alterations in crustacean body colors [22]. Overall, research on the impacts of photoperiod on stress responses, body color changes, and corresponding physiological and biochemical reactions in crustaceans remains relatively limited.

Photoperiod induces a series of physiological and biochemical activities within an animal while also regulating its growth and development. For instance, studies on Fugu rubripes (*Takifugu rubripes*) revealed that the growth of larvae after 19 days of hatching was enhanced under conditions of 20 h of daylight exposure each day [23]. Conversely, reduced duration of light exposure in the photoperiod accelerated the development and metamorphosis rate of green frog (*Rana clamitans*) and gray treefrog (*Hyla versicolor*) tadpoles [24]. The growth of crustaceans often coincides with their molting behavior. Each molt results in increased size and weight, accompanied by complex physiological regulatory processes [19,25]. The retinoid X receptor gene (*RXR*) plays a significant role in regulating molting in crustaceans [26]. An increase in the expression level of *RXR* implies a higher likelihood of molting [11]. In addition, α-amylase exhibits a strong correlation with the digestive capacity and nutritional metabolism of animals, directly reflecting their digestive absorption ability. The expression level of the α-amylase gene (*α-AMY*) can reflect the growth status of the animal [27]. Therefore, these two genes related to molting and growth in crustaceans could indicate their growth conditions. However, the current research on the photoperiod regulation of the *RXR* and *α-AMY* genes in crustaceans remains limited.

Redclaw crayfish (*Cherax quadricarinatus*), known for its rapid growth, good meat quality, and significant economic potential, is one of the most promising freshwater shrimp species in aquaculture. According to the 2021 FAO statistics, countries such as Australia, Cambodia, Malaysia, Indonesia, etc., had a total production of approximately 260 tons of *C. quadricarinatus* [28]. The results from a 2023 industry survey conducted by our collaborating companies indicate that the annual total production of *C. quadricarinatus* in China is currently around 20,000 tons, with a market selling price of about USD 20 per kilogram for mature crayfish weighing between 50 g and 100 g. The reproduction, growth, and survival of *C. quadricarinatus* juveniles are significantly influenced by environmental factors. Under conventional farming conditions, unexplained mass mortality often occurs. To enhance the productivity of species, it is crucial to determine the optimal parameters for *C. quadricarinatus* juveniles in response to various environmental factors. Although there have been reports on environmental factors that affect the developmental stages of *C. quadricarinatus*, only one study has reported the effect of photoperiod on newly hatched *C. quadricarinatus* juveniles. The study was limited to a culture period of 14 days, compared to the expected industrial cultural period of approximately 30 days for *C. quadricarinatus* (with a body size range of 3–5 cm). Therefore, further improvements are necessary in this research [19]. To address the existing research gap, this study investigated the effects of different photoperiods on *C. quadricarinatus* juveniles over a 30-day culture period, focusing on survival, growth performance, enzyme activities, body color, and growth-related gene expression. The objective of this research was to establish a foundation for understanding the photoperiod regulation of physiological and biochemical responses in *C. quadricarinatus* juveniles.

## 2. Materials and Methods

### 2.1. Ethics Statement

This study received approval from the Animal Experiment Ethics Committee of the Pearl River Fisheries Research Institute, Chinese Academy of Fishery Sciences (Approval No. LAEC-PRFRI-2023-05-01; Approval Date: 1 May 2023). Effort was made to minimize the pain experienced by the *C. quadricarinatus* during the study.

### 2.2. Experimental Methods

#### 2.2.1. Preparation of the Culture Environment

The tanks used for the experiment were cleaned and disinfected and filled with pre-aerated recirculating water (Figure S1). The dissolved oxygen levels ranged from 7.25 to 8.66 mg/L, pH ranged from 7.80 to 8.27, and temperature was maintained at 30 ± 0.5 °C. Nontoxic water quality improver tablets (Xiande Biological Technology Co., Ltd., Guangzhou, China) were added to the filtration tank to enhance water quality. The aeration system was cleaned with chlorine-containing water, and the residual chlorine in each tank was measured after 24 h. The experimental juveniles were introduced when the residual chlorine was below 0.005 mg/L. Similar environmental conditions were maintained for all experimental groups during the acclimation period.

#### 2.2.2. Experimental Design

A total of 510 healthy 7-day-old *C. quadricarinatus* juveniles (initial body length of 1.06 ± 0.14 cm, initial body weight of 0.03 ± 0.01 g) were obtained from Hengzhao Lanlong Aquaculture Co., Ltd. (Jiangmen, China). Before the experiment, newly hatched juveniles were transferred to the Pearl River Fisheries Research Institute and acclimatized for 1 week in aquariums (length = 130 cm, width = 52 cm, height = 60 cm), with a stocking density of 50 individuals per square meter. The exterior surface of all the aquariums was covered with black plastic film to prevent external light interference. The water depth in the aquariums was 45 cm. Each tank was equipped with custom-made lighting fixtures with 18 W white LED lights placed 30 cm above the water surface to maintain a light intensity of 1000 lx below the water surface. Five different photoperiod experimental groups were set up: 0L:24D (constant darkness), 6L:18D (lights on at 12:00, off at 18:00), 12L:12D (lights on at 06:00, off at 18:00), 18L:6D (lights on at 06:00, off at 24:00), and 24L:0D (constant light). Each group had 3 replicates, with 34 individuals per replicate, and the rearing period lasted for 30 days. The timing of light on/off was controlled by Pinyi al-06 automatic timers (Pinyi Electric Appliance, Inc., Ningbo, China).

#### 2.2.3. Daily Management

Commercial shrimp feed (containing 32% crude protein, 12% crude fat, and 9% moisture) was offered twice daily (at 08:00 and 17:00) to the experimental juveniles. One hour after each feeding, siphon out the remaining feed, and replace one-fifth of the water every two days. Water samples were collected daily. Water quality parameters such as pH, ammonia nitrogen, nitrite, dissolved oxygen, residual chlorine, sulfide, and total alkalinity were measured using an Octadem W-II water quality tester (Octadem Technology, Inc., Wuxi, China) and its accompanying reagents to ensure good water quality. The water used in the experiment was continuously aerated and maintained according to actual production standards. The temperature and light intensity of the rearing water were periodically measured using a TASI TA8121 illuminometer (TASI Electronics, Inc., Suzhou, China).

### 2.3. Sample Collection and Analysis

#### 2.3.1. Growth and Survival Indices

After 30 days of cultivation, 10 juveniles were randomly selected from each group. The body length and weight of each group were measured according to the method in our previous study [21] using tpsDig2 software version 1.40 (F. James Rohlf, Stony Brook University, Stony Brook, NY, USA) and a Mettler Toledo AL-204 precision balance (Mettler Toledo, Inc., Shanghai, China). In addition, 4–6 juveniles were randomly selected from each aquarium. Hemolymph, eyestalk, and hepatopancreas tissues were collected from these juveniles, immediately frozen in liquid nitrogen, and stored at −80 °C for subsequent analysis of antioxidant properties, immune capacities, and melatonin and cortisol levels. Each sample underwent three repeated tests. Growth-related parameters were calculated as follows:

(1) $$\mathrm{Survival}\;\mathrm{rate}\;(\%)=\frac{N_{t}}{N_{0}}\times 100\%$$

(2) $$\mathrm{Weight}\;\mathrm{gain}\;\mathrm{rate}\;WGR(100\%)=\frac{W_{f}-W_{i}}{W_{i}}\times 100\%$$

(3) $$\mathrm{Total}\;\mathrm{length}\;\mathrm{gain}\;\mathrm{rate}\;TLGR(100\%)=\frac{L_{f}-L_{i}}{L_{i}}\times 100\%$$

(4) $$\mathrm{Specific}\;\mathrm{growth}\;\mathrm{rate}\;SGR(100\%)=\frac{\ln W_{f}-\ln W_{i}}{t}$$

where N_0_ and N_t_ are the initial and final number of individuals, respectively; W_i_ and W_f_ are the initial and final weights, respectively (g); L_i_ and L_f_ are the initial and final length, respectively (cm).

#### 2.3.2. Determination of Enzyme Activity and Hormone Levels

The collected hepato-pancreatic samples underwent antioxidant and immune enzyme activity assays. Superoxide dismutase (SOD, A001-3-2), malondialdehyde (MDA, A003-1-2), acid phosphatase (ACP, A060-1-1), and lysozyme (LZM, A050-1-1) activities were determined using the WST-1 method, thiobarbituric acid (TBA) reaction, spectrophotometry, and turbidimetry, respectively. Procedures were conducted following the respective commercial kit instructions (Nanjing Jiancheng Bioengineering Institute, Nanjing, China). Blood lymph and eyestalk were mixed separately with PBS (*w*:*v* = 1:9), homogenized in liquid-nitrogen-containing glass mortars, and then centrifuged at 5000× *g* for 5 min at 4 °C to collect the supernatant for further use. ELISA assay kits for melatonin and cortisol (Catalog No. YJ093369; YJ085236) (Shanghai Jichun Industry Co., Ltd., Shanghai, China) were employed. Standard curves for melatonin and cortisol were generated using six dilution series: 0, 5, 10, 20, 40, and 80 pg mL^−1^ and 0, 12.5, 25, 50, 100, and 200 ng mL^−1^, respectively. Based on the competitive binding principle, the determination was carried out on microtiter plates, where melatonin or cortisol from standard solutions competed with those combined with horseradish peroxidase for antibody binding sites. The plates were incubated at 37 °C for 30 min, and unbound components were washed away using a washing solution. The content of the bound melatonin or cortisol enzyme conjugate was determined via the reaction of peroxidase with the substrate tetramethylbenzidine (TMB) at 37 °C for 15 min (stopped by adding 100 μL 0.5 M H_2_SO_4_), and the absorbance was read at 450 nm using an absorbance Microplate Reader (SpectraMax 190, Molecular Devices, San Jose, CA, USA).

#### 2.3.3. Body Color Measurement

Following the experiment, a 3nh high-precision colorimeter (Shenzhen ThreeNH Technology Co., Ltd., Shenzhen, China) was employed to measure the brightness (L*), red–green (a*), and yellow–blue values (b*) of the dorsal carapace in each group of *C. quadricarinatus* (Figure S2). A higher L* value signifies a brighter color and lighter body color, whereas a lower L* value indicates a darker color and deeper body color. A positive a* value represents a reddish tone, whereas a negative value signifies greenish. Similarly, a positive b* value indicates a yellowish tone, whereas a negative value implies a bluish hue.

To compare the colors of *C. quadricarinatus* juveniles under different photoperiods, the CIEDE2000 formula was used to calculate the color difference (ΔE):

(5) $$\Delta E=\sqrt{{\left(L^{*}-L^{*\prime }\right)}^{2}+{\left(a^{*}-a^{*\prime }\right)}^{2}+{\left(b^{*}-b^{*\prime }\right)}^{2}}$$

where L*, a*, and b* are from one photoperiod and L*′, a*′, and b*′ are from another photoperiod.

#### 2.3.4. Total RNA Extraction, cDNA Synthesis, and qPCR Analysis

Total RNA was extracted from the hepato-pancreas using Trizol Reagent (Invitrogen, Waltham, MA, USA). Its integrity was tested via 1% agarose gel electrophoresis and quantified for concentration and purity using a NanoDrop-2000 spectrophotometer. Subsequently, DNase I enzyme (New England Biolabs, Ipswich, MA, USA) was used to digest total RNA (1 µg) for 15 min. The M-MLV reverse transcriptase kit (Invitrogen, Waltham, MA, USA) was used to synthesize cDNA from 1 µg total RNA for each sample according to the instructions in the kit.

The primers for target and reference genes used in this study are listed in Table 1. Real-time fluorescence quantitative PCR (qPCR) was performed using the Step One Plus Real-time PCR system (Applied Biosystems, Foster City, CA, USA) to evaluate the expression levels of *RXR* and *α-AMY* genes in *C. quadricarinatus* juveniles under different photoperiods. Each sample underwent three repeated tests. Each qPCR contained 1 µL (50 ng/µL) cDNA, 5 µL iTaq™ Universal SYBR^®®^ Green Supermix, 0.5 µL of each pair of primers (10 pmol/µL), and 3 µL double-distilled water, forming a final volume of 20 µL. To detect the expression levels of each target gene, the reaction procedures were as follows: 95 °C for 3 min; 35 cycles of 95 °C for 40 s, 60 °C for 45 s, 72 °C for 30 s, and 72 °C for 10 min; and 95 °C for 5 s, 60 °C for 30 s, and 95 °C for 15 s.

### 2.4. Statistical Analysis

GraphPad Prism version 8.0.2 for Windows (GraphPad Software, Boston, MA, USA) was utilized to assess the normality distribution and uniformity (Shapiro–Wilk) of the experimental data, ensuring the data’s validity. All data were presented as mean ± SD. After arc-sine transformation, a one-way analysis of variance (ANOVA) was performed on each set of experimental data to identify intergroup differences. Tukey’s multiple comparison test served as a post hoc analysis, with statistical significance set at *p* < 0.05.

## 3. Results

### 3.1. Effects of Different Photoperiod Treatments on Survival Rate and Growth of C. quadricarinatus Juveniles

The results from the five treatment groups (Table 2) indicate that the survival rates of *C. quadricarinatus* juveniles in the 18L:6D and 24L:0D groups were significantly higher than those in the 6L:18D and 12L:12D groups (*p* < 0.05) and markedly higher than that in the 0L:24D group (*p* < 0.01). Moreover, the final body weight and weight gain rate of *C. quadricarinatus* juveniles in the 0L:24D group were significantly higher than those in the 12L:12D, 18L:6D, and 24L:0D groups (*p* < 0.05), but no significant differences were detected between the 6L:18D group and other groups (*p* > 0.05). In addition, the final body length of *C. quadricarinatus* juveniles in the 0L:24D group was significantly higher than that in the 12L:12D group, but no significant differences were observed among other groups (*p* > 0.05). There were no significant differences in the body length growth rate of *C. quadricarinatus* juveniles among different photoperiod groups (*p* > 0.05). Furthermore, the SGR of *C. quadricarinatus* juveniles in the 0L:24D group was significantly higher than those in the 12L:12D and 18L:6D groups, but no significant differences were observed among the other groups (*p* > 0.05).

### 3.2. Effects of Different Photoperiod Treatments on Antioxidative Capacity and Immune-Related Enzyme Activity of C. quadricarinatus Juveniles

#### 3.2.1. Antioxidant Indices in Different Photoperiod Groups

**Figure 1:**
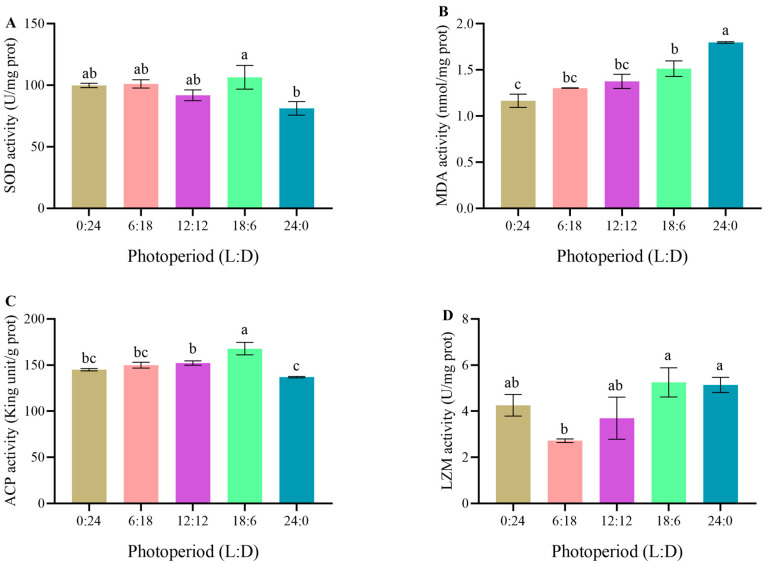
Related enzyme activities of *Cherax quadricarinatus* after 30 days of culture under five photoperiod regimes ((**A**): SOD; (**B**): MDA; (**C**): ACP; (**D**): LZM). The data are expressed as mean ± SD of three replicates (*n* = 9). Different letters indicate significant differences (LSD; *p* < 0.05). L: light, D: dark.

The 18L:6D group had the highest SOD activity, which was significantly higher than that in the 24L:0D group (*p* < 0.05), but showed no significant differences among other groups (*p* > 0.05) (Figure 1A). With the extension of daylight exposure, MDA content gradually increased. The MDA content in the 24L:0D group was significantly higher than those in the 6L:18D, 12L:12D, and 18L:6D groups (*p* < 0.05) and markedly higher than that in the 0L:24D group (*p* < 0.01). In addition, the MDA content in the 18L:6D group was significantly higher than that in the 0L:24D group (*p* < 0.05) but showed no significant differences from those in other groups (*p* > 0.05) (Figure 1B).

#### 3.2.2. Immune Enzyme Activities in Different Photoperiod Groups

The ACP activity in the 18L:6D group was the highest; it was significantly higher than those in the 0L:24D, 6L:18D, and 12L:12D groups (*p* < 0.05) and markedly higher than that in the 24L:0D group (*p* < 0.01). In addition, the ACP activity in the 12L:12D group was significantly higher than that in the 0L:24D group (*p* < 0.05) but showed no significant differences from those in other groups (*p* > 0.05) (Figure 1C). The LZM activities in the 18L:6D and 24L:0D groups were significantly higher than that in the 6L:18D group (*p* < 0.05) but showed no significant differences from those in other groups (*p* > 0.05) (Figure 1D).

### 3.3. Effects of Different Photoperiod Treatments on Melatonin and Cortisol in C. quadricarinatus Juveniles

**Figure 2:**
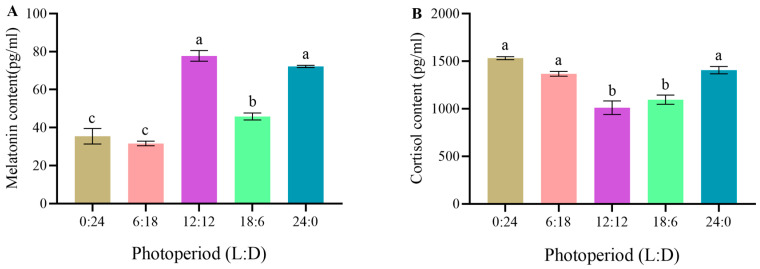
Melatonin and cortisol levels in *C. quadricarinatus* after 30 days of culture under five photoperiod regimes ((**A**): melatonin; (**B**): cortisol). The data are expressed as mean ± SD of three replicates (*n* = 9). Different letters indicate significant differences (LSD; *p* < 0.05). L: light, D: dark.

The melatonin levels in the 12L:12D and 24L:0D groups were significantly higher than that in the 18L:6D group (*p* < 0.05) and markedly higher than those in the 0L:24D and 6L:18D groups (*p* < 0.01). In addition, the melatonin level in the 18L:6D group was significantly higher than those in the 0L:24D and 6L:18D groups (*p* < 0.05) (Figure 2A). Concerning cortisol, the levels in the 0L:24D, 6L:18D, and 24L:0D groups were significantly higher than those in the 12L:12D and 18L:6D groups (*p* < 0.05), but no significant differences were observed among the other groups (*p* > 0.05) (Figure 2B).

### 3.4. Effects of Different Photoperiod Treatments on Body Color Changes in C. quadricarinatus Juveniles

**Figure 3:**
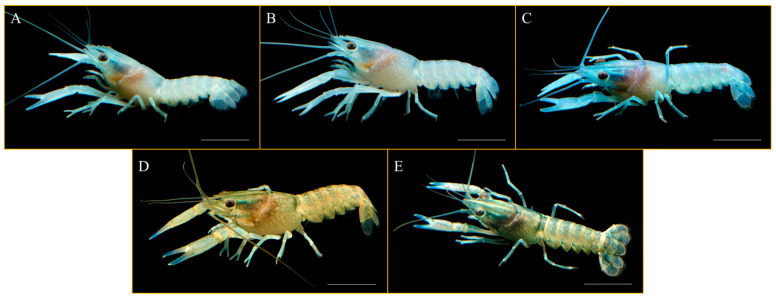
Body color of *C. quadricarinatus* after 30 days of cultivation under different photoperiods ((**A**): 0L:24D; (**B**): 6L:18D; (**C**): 12L:12D; (**D**): 18L:6D; (**E**): 24L:0D). Scale bar = 1 cm.

Through visual observation, distinct differences in body color were observed in *C. quadricarinatus* juveniles cultured under different photoperiods for 30 days. With an increase in daylight exposure, the body color of the juveniles gradually darkened. Specifically, the juveniles cultured under the 0L:24D, 6L:18D, and 12L:12D photoperiods had a light blue color, whereas those under the 18L:6D and 24L:0D photoperiods displayed a yellow–brown color (Figure 3).

**Figure 4:**
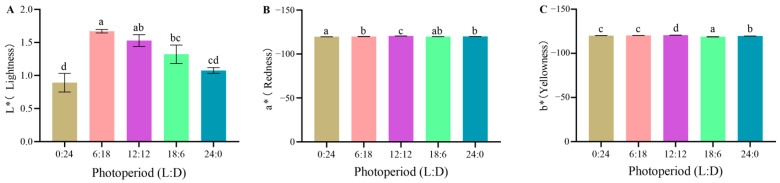
L*, a*, and b* of *C. quadricarinatus* after 30 days of culture under five photoperiod regimes ((**A**): L*; (**B**): a*; (**C**): b*). The data are expressed as mean ± SD of three replicates (*n* = 9). Different letters indicate significant differences (LSD; *p* < 0.05). L: light, D: dark.

Instrumental measurements revealed that the L*, a*, and b* values of juvenile body color after 30 days of culture varied. The L* value was the highest in the 6L:18D group (1.67); however, it was not significantly different from that in the 12L:12D group (*p* > 0.05) but significantly different from those in the other three groups (*p* < 0.05). The lowest L* value was recorded in the 0L:24D group (0.89) (Figure 4A). The a* value was the highest in the 0L:24D group; however, it was not significantly different from that in the 18L:6D group (*p* > 0.05) but significantly different from those in the other groups (*p* < 0.05). The 12L:12D group had the lowest a* value, which was significantly different from those in the other groups (*p* < 0.05) (Figure 4B). In addition, the b* value was the highest in the 18L:6D group and was significantly higher than those in the other groups (*p* < 0.05). Moreover, the 12L:12D group had the lowest b* value, which was significantly lower than those in the other groups (*p* < 0.05) (Figure 4C).

Color differences among *C. quadricarinatus* juveniles cultured under different photoperiods were observed to varying extents (Table 3). The greatest color difference was found between the 0L:24D and 24L:0D groups (ΔE = 2.02), whereas the least color difference was found between the 0L:24D and 6L:18D groups (ΔE = 0.53).

### 3.5. Effects of Different Photoperiod Treatments on the Expression of Growth-Related Genes in C. quadricarinatus Juveniles

**Figure 5:**
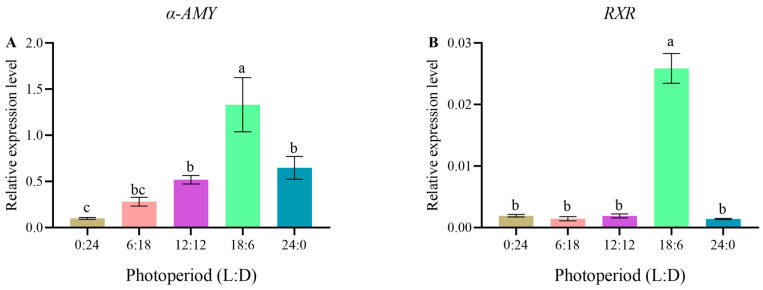
Related genes’ relative expression level of *C. quadricarinatus* after 30 days of culture under five photoperiod regimes ((**A**): *RXR*; (**B**): *α-AMY*). The data are expressed as mean ± SD of three replicates (*n* = 9). Different letters indicate significant differences (LSD; *p* < 0.05). L: light, D: dark.

qRT-PCR results revealed that the highest relative expression level of the *α-AMY* gene was recorded in the 18L:6D group, and it was significantly higher than those in the 6L:18D, 12L:12D, and 24L:0D groups (*p* < 0.05) and markedly higher than that in the 0L:24D group (*p* < 0.01). In addition, the expression levels of the *α-AMY* gene in the 12L:12D and 24L:0D groups were significantly higher than that in the 0L:24D group (*p* < 0.05) but showed no significant differences with those in the other groups (*p* > 0.05) (Figure 5A). Furthermore, the relative expression level of the *RXR* gene in the 18L:6D group was significantly higher than those in all the other groups (*p* < 0.01) but showed no significant differences with the other groups (*p* > 0.05) (Figure 5B).

## 4. Discussion

### 4.1. Effects of Different Photoperiod Treatments on Survival Rate and Growth of C. quadricarinatus Juveniles

Physiological activities such as survival and growth in crustaceans are directly or indirectly influenced by photoperiod [29,30]. For instance, when daylight exposure exceeds 12 h, the survival rate of *M. rosenbergii* juveniles increases, and, with prolonged exposure, the metamorphosis rate of the juveniles gradually increases [21]. In addition, zoea larva of swimming crab (*Portunus trituberculatus*) failed to metamorphose under constant darkness, 6 h of daylight, or constant light conditions; however, the survival and metamorphosis rates of the zoea larva under 12–18 h of daylight were high and indicated better growth performance [3]. In this study, *C. quadricarinatus* juveniles showed higher survival rates under 18L:6D and 24L:0D photoperiod treatments and the lowest survival rates under the 0L:24D photoperiod. However, Cheng et al. [19] reported contrasting findings in which *C. quadricarinatus* juveniles showed lower survival rates under 18L:6D and 24L:0D photoperiods and higher survival rates under the 0L:24D photoperiod. Despite using newly hatched *C. quadricarinatus* juveniles as we did in this study, their [19] shorter experimental duration of only 14 days might explain the instability in the survival rates during the crucial molting and growth phases of the initial 2 weeks post-hatch. In these phases, the survival rate of *C. quadricarinatus* juveniles was greatly influenced by external factors, leading to unstable survival rates. In contrast, we acclimated newly hatched *C. quadricarinatus* juveniles in a recirculating water system for 1 week before starting the experiment. This adaptation period potentially enhanced their suitability for the rearing environment, leading to more stability in survival rates. Furthermore, the rearing period in our experiment was 30 days, which is consistent with practices in industrial-scale production. Moreover, Wu et al. [31] reported that, under 18L:6D and 24L:0D photoperiods, *C. quadricarinatus* juveniles exhibited higher survival rates, whereas, under the 0L:24D photoperiod, the juveniles exhibited the lowest survival rates, and this is consistent with our findings.

Although the survival rate of *C. quadricarinatus* under the 0L:24D photoperiod was low, the weight gain rate and final body weight were high. According to preliminary results, the stocking density of *C. quadricarinatus* juveniles used in our experiment was only 50 individuals per m^2^. At this density, intraspecific aggression and competition do not significantly affect the survival and growth rate of juveniles. However, as the experiment progressed, variations in individual sizes of *C. quadricarinatus* increased, escalating aggressive behavior and cannibalism. Furthermore, the behavior of crustaceans is mostly rhythmic; they typically hide underwater during the day and forage at night [32]. Complete darkness has been inferred to facilitate the activity of *C. quadricarinatus* juveniles, increasing the feeding frequency and intake, which in turn enhances weight gain. In contrast, as daylight exposure increased, the inhibitory effect on the activity of *C. quadricarinatus* juveniles decreased intraspecific competition and aggression, thereby improving survival rates. Overall, based on our findings, 18–24 h of light exposure per day favors the health of *C. quadricarinatus* juveniles and increases their survival rate.

### 4.2. Effects of Different Photoperiod Treatments on Antioxidative Capacity and Immune-Related Enzyme Activity of C. quadricarinatus Juveniles

The production of ROS is a primary cause of various oxidative damages in aquatic organisms [33]. These free radicals can lead to DNA damage, lipid peroxidation, and protein carbonylation and serve as sensitive biomarkers of cellular response to pollutant exposure [34]. Changes in photoperiod induce variations in the level of ROS within crustaceans, ultimately resulting in oxidative stress and triggering immune response reactions within the organism [19,33]. In this process, SOD plays a crucial role in antioxidant defense by neutralizing ROS and reducing lipid peroxidation damage. MDA is the oxidation end product from the action of free radicals on lipids within an organism. It can lead to cross-linking polymerization of significant biomolecules such as proteins and nucleic acids and possesses a certain degree of cellular toxicity. Therefore, MDA level is often employed as an indicator of cell membrane damage caused by lipid peroxidation [34]. Relevant studies on *P. trituberculatus* showed that reduced SOD activity and elevated MDA levels adversely affected redox balance, exerting a negative impact on the normal physiological metabolism of crabs [35]. In the present study, the SOD activity of *C. quadricarinatus* juveniles was high under the 18L:6D photoperiod but low under the 24L:0D photoperiod, accompanied by the highest MDA level. Therefore, it is speculated that 18 h of light exposure per day is conducive to maintaining the oxidative balance of *C. quadricarinatus* juveniles and is more effective in disease prevention compared to continuous illumination.

Crustaceans are different from vertebrates because they lack acquired specific immune functions but possess a robust nonspecific immune system that swiftly identifies and effectively clears invading microorganisms [16]. LZM, an antimicrobial peptide, primarily protects the stability of organisms by hydrolyzing the cell walls of Gram-positive bacteria and removing foreign harmful substances. It contributes to the nonspecific immune response in crustaceans [36]. Environmental stress often triggers nonspecific immune reactions in aquatic animals. ACP participates in phosphoric acid hydrolysis during metabolism and lysosomal digestion of invading organisms, playing a significant role in immune defense and recognition in crustaceans [37]. In the present study, ACP and LZM activities were relatively high under an 18L:6D photoperiod, indicating strong immune capability and good survival rates in juveniles. However, studies on *P. vannamei* and *M. rosenbergii* have revealed that ACP and LZM activities in *P. vannamei* juveniles were higher under 2–4 h of daylight exposure compared to longer illumination periods [16], whereas the LZM activity in *M. rosenbergii* juveniles was the lowest under the 18L:6D photoperiod [21]. These results are not consistent with the findings of our study. Furthermore, studies indicated that LZM activity in Dybowski’s frog (*Rana dybowskii*) increased in dark conditions and decreased with prolonged exposure to daylight [38], but LZM activity in *T. rubripes* larvae remained unaffected by photoperiod changes [23]. Therefore, the influence of photoperiod on ACP and LZM activity in aquatic organisms varies across different species.

### 4.3. Effects of Different Photoperiod Treatments on Melatonin and Cortisol in C. quadricarinatus Juveniles

Organisms transmit photoperiod signals internally through biogenic amines, particularly melatonin [39]. Melatonin is highly conservative, as evidenced by its widespread distribution across prokaryotic and eukaryotic organisms and ubiquitous molecular structure [40]. Melatonin plays an important role in light perception and signal transduction of animals, acting as a chemical messenger that conveys photoperiodic information to the organism [41]. As a result, the level of melatonin within an organism is subject to regulation by circadian rhythms [17]. In the present study, *C. quadricarinatus* juveniles reared under different photoperiods had partially dissimilar levels of melatonin. This discrepancy could be explained by the phase shift in melatonin rhythm. Changes in photoperiod impact both the rhythm of melatonin and the duration of nighttime melatonin levels [42,43]. Moreover, melatonin possesses antioxidant properties, which help to reduce oxidative stress in crustacean tissues [17]. Extensive research has validated the integral role of melatonin in the antioxidant defense system, highlighting its regulatory influence on certain antioxidant enzymes in organisms. For instance, both SOD and glutathione peroxidase (GSH-Px) serve as scavengers for ROS [44,45,46,47]. Therefore, melatonin acts as a direct scavenger of free radicals and is capable of neutralizing both reactive oxygen and nitrogen species. It constitutes a vital component of the antioxidant defense mechanism of organisms. In the present study, the melatonin levels were higher under the 12L:12D and 24L:0D photoperiods compared to that in the 18L:6D group. However, the 18L:6D group exhibited the highest SOD activity and the most robust antioxidant capacity, whereas the 0L:24D and 6L:18D groups displayed the lowest melatonin levels, reduced antioxidant capacity, and low survival rates. Hence, excessively high or low levels of melatonin in *C. quadricarinatus* juveniles may negatively impact their antioxidant defense. Appropriate melatonin levels strengthen the antioxidant capability of these juveniles. Geihs et al. [48] and Maciel et al. [49] injected melatonin into *N. granulata*, and their experiments showed that melatonin participated in the antioxidant defense mechanism of the crab muscles and gills, and a proper level of melatonin could enhance the antioxidant defense in *N. granulata*, corroborating the results of our research findings.

Cortisol is a major indicator used in assessing the internal homeostasis of aquatic organisms. It participates in various physiological processes, including lipid metabolism, stress response, and osmotic regulation [50]. As the principal stress hormone in animals, elevated plasma cortisol levels in peripheral blood are often considered indicative of stress [14]. Studies suggest that artificially controlled photoperiods are a chronic stressor. Shortened or prolonged duration of light exposure may induce stress in crustaceans, which can be assessed through increased cortisol levels [20,51]. In the present study, as light exposure duration gradually increased from 0 to 12 h, *C. quadricarinatus* cortisol levels decreased incrementally, reaching the lowest point at 12 h of light exposure. Subsequently, as the duration extended from 12 to 24 h, *C. quadricarinatus* cortisol levels gradually increased. Leonardi and Klempau [52] previously demonstrated that artificial photoperiods not only induce robust stress responses in rainbow trout (*Oncorhynchus mykiss*) but also maintain high plasma cortisol levels for up to two months following the cessation of the artificial regulation. Our results demonstrate that, compared to a natural photoperiod, alteration in the duration of daylight exposure, either by shortening or prolonging it, induces stress in *C. quadricarinatus* juveniles, consequently elevating their cortisol levels. This suggests that changes in photoperiod exert a stress response in the physiology of *C. quadricarinatus*.

### 4.4. Effects of Different Photoperiod Treatments on Body Color Changes in C. quadricarinatus Juveniles

Several animals change their body color to serve various functions, including thermoregulation [53], camouflage defense [54], mate attraction [55], and identification and communication [56]. Studies have shown that changes in photoperiod can affect the body color of certain aquatic animals. For example, mature Atlantic salmons (*Salmo salar*) exhibit a more rapid transition from dark skin to silver skin when exposed to constant light conditions as opposed to varying light conditions [57]. African catfish (*Clarias gariepinus*) raised in total darkness have darker skin colors in comparison to those raised in fully illuminated conditions [58]. In the present study, the extension of daylight exposure gradually intensified the body color of *C. quadricarinatus* juveniles from pale blue to yellow–brown. This observation shows that varying photoperiods can induce changes in the body color of *C. quadricarinatus* during growth. Animal body color is influenced by pigment deposition. Crustaceans lack the ability to synthesize pigments; they acquire pigments such as carotenoids through dietary intake and further deposit and transform them into the required pigment types [59,60]. The blue color in crustaceans is associated with the product crustacyanin formed from the binding of protein and astaxanthin [61,62,63]. Research on the impact of adding astaxanthin to the feed of *P.clarkii* has shown that supplementation with astaxanthin deepens the coloration of the crayfish. Conversely, a deficiency in astaxanthin results in a weakening of the *P.clarkii* coloration, causing it to exhibit a pale blue hue [64]. Studies on the effects of different environment color on *C. quadricarinatus* body color showed that juveniles reared in white, red, green, or blue environments displayed yellow–brown color with high astaxanthin content, whereas those raised in a black environment exhibited deep blue color with low astaxanthin content [65]. Therefore, it is speculated that daylight exposure below 12 h is detrimental to astaxanthin production in *C. quadricarinatus*, resulting in a bluish color, whereas exposure durations between 18 and 24 h promote astaxanthin synthesis and cause the body color of *C. quadricarinatus* to shift to yellow–brown.

### 4.5. Effects of Different Photoperiod Treatments on the Expression of Growth-Related Genes in C. quadricarinatus Juveniles

The regulatory mechanism underlying the physiological activities of animal growth and metabolism has been a focal point in biological research. The expression levels of growth- and development-related genes are closely associated with the growth of an organism [66,67]. Among the genes that influence animal growth, *α-AMY* gene expression level reflects the digestive absorption and nutritional metabolic capabilities of organisms [68]. Moreover, α-amylase is the expression product of the *α-AMY* gene, serving as an endo-amylolytic enzyme. By catalyzing the hydrolysis of α-1,4-glycosidic bonds, it facilitates the degradation of starch and glycogen in animals into smaller compounds such as glucose and maltose, thereby promoting digestive absorption [69]. The significance of the *α-AMY* gene extends to crustaceans as it exhibits a high correlation with their digestive absorption and metabolic processes. The gene has been considered to be a candidate gene for studying crustacean growth and development [66]. In this study, the highest expression level of the *α-AMY* gene in *C. quadricarinatus* juveniles was at 18 h of daylight exposure but decreased under constant light. This suggests that appropriate extension of the duration of daylight exposure may enhance the digestive absorption capability of *C. quadricarinatus*. Crustaceans are aquatic organisms that rely on molting as a crucial process for growth. Molting is a vital aspect of the life cycle of crustaceans. This shedding process is mainly regulated by intricate interactions among endogenous hormones in these animals [67]. *RXR*, widely present in crustaceans, is a highly conserved member of the nuclear receptor superfamily. During crustacean molting, ecdysteroids are converted to 20-hydroxyecdysone (20E). The latter is transported via the hemolymph to target cells and binds to heterodimeric complexes formed by *RXR* and *EcR*, thereby initiating a cascade of reactions to regulate molting [70,71]. Thus, the *RXR* gene is considered among the candidate genes related to crustacean growth. In the present study, the highest expression of the *RXR* gene in *C. quadricarinatus* juveniles was under the 18L:6D photoperiod, suggesting that the high expression of this gene under this condition promotes molting in juveniles. However, the specific regulatory mechanism requires further investigation.

## 5. Conclusions

This study demonstrated that, under an 18L:6D photoperiod, *C. quadricarinatus* juveniles exhibited the highest survival rate, relatively good growth performance, strong antioxidant stress response, and good immune defense capabilities. In contrast, under a 0L:24D photoperiod, the juveniles exhibited low survival rates, reduced antioxidant stress response, and decreased immune enzyme activity. Therefore, this study confirms that the 18L:6D photoperiod is advantageous for industrialized cultivation of *C. quadricarinatus* juveniles, whereas the dark (0L:24D) cultivation condition is unfavorable for their industrial cultivation. This study also showed that the body color of *C. quadricarinatus* juveniles gradually changed from light blue to yellow–brown with increasing light exposure duration.

## Figures and Tables

**Table 1:** Primers used for qPCR in this study.

Gene	Sequence (5′–3′)	Sources
*RXR*	F: AGGAGATGCCGTAACCAACA	KM016907.1
	R: ATGCTTCGGTGTGAGAAGGA	
*α-AMY*	F: CCGCTGGAGACAGATCTACG	OL963595.1
	R: AACGTCACAGTAGGTGCCAG	
*β-actin*	F: CCCCATGCTATCTTGCGTCT	MN396754.1
	R: CGTCAGGAAGCTCGTAGGAT	

**Table 2:** Growth indexes of *Cherax quadricarinatus* juveniles under different photoperiods.

Photoperiod (L:D)	Survival Rate (%)	Final Body Weight (g)	Weight Gain Rate (%)	Final Full Length (cm)	Total Length Gain Rate (%)	SGR (%)
0:24	35.67 ± 2.52 ^c^	0.96 ± 0.21 ^a^	2822.00 ± 168.50 ^a^	2.96 ± 0.13 ^a^	180.00 ± 22.54	0.11 ± 0.00 ^a^
6:18	49.67 ± 3.51 ^b^	0.64 ± 0.13 ^ab^	2093.00 ± 317.90 ^ab^	2.68 ± 0.16 ^ab^	154.00 ± 22.65	0.10 ± 0.01 ^ab^
12:12	54.17 ± 3.40 ^b^	0.48 ± 0.07 ^b^	1574.00 ± 100.80 ^b^	2.37 ± 0.14 ^b^	144.10 ± 20.56	0.09 ± 0.00 ^b^
18:6	74.17 ± 3.69 ^a^	0.53 ± 0.02 ^b^	1745.00 ± 306.50 ^b^	2.78 ± 0.09 ^ab^	163.70 ± 26.08	0.10 ± 0.01 ^b^
24:0	66.83 ± 2.26 ^a^	0.51 ± 0.17 ^b^	1721.00 ± 373.70 ^b^	2.86 ± 0.30 ^a^	169.30 ± 16.29	0.10 ± 0.00 ^ab^

All data are presented as the mean ± SD. Different superscript letters represent statistically significant differences (*p* < 0.05).

**Table 3:** Matrix of pair-wise ΔE values among experimental groups of *C. quadricarinatus*.

Photoperiod (L:D)	0:24	6:18	12:12	18:6
6:18	0.53	——	——	——
12:12	0.82	0.78	——	——
18:6	1.41	1.21	0.95	——
24:0	2.02	1.55	1.23	0.84

## Data Availability

The data presented in this study are available on request from the corresponding author.

## Supplementary Materials

The following supporting information can be downloaded at https://www.mdpi.com/article/10.3390/ani14030411/s1, Figure S1: Experimental water tank; Figure S2: The red box represents the region for *C. quadricarinatus* color detection.
