# Supplementary material for: Effects of Photoperiod on Survival, Growth, Physiological, and Biochemical Indices of Redclaw Crayfish (Cherax quadricarinatus) Juveniles
Source: Animals (Basel). 2024 Jan 26;14(3):411. doi: 10.3390/ani14030411 (PMC10854630; doi:10.3390/ani14030411)

**Supplementary Material**

**Figure S1.** Experimental water tank.

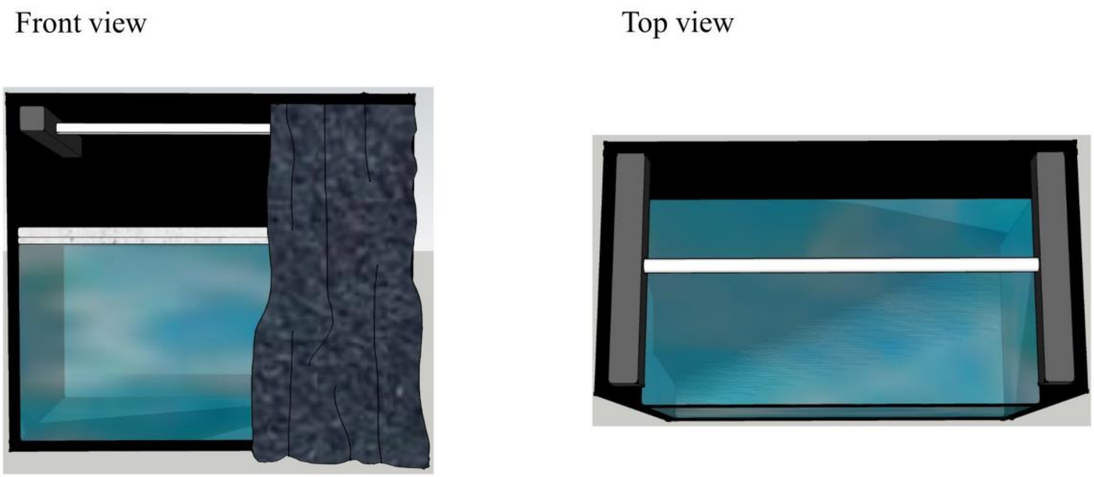


**Figure S2.** The red box represents the region for *C. quadricarinatus* color detection.

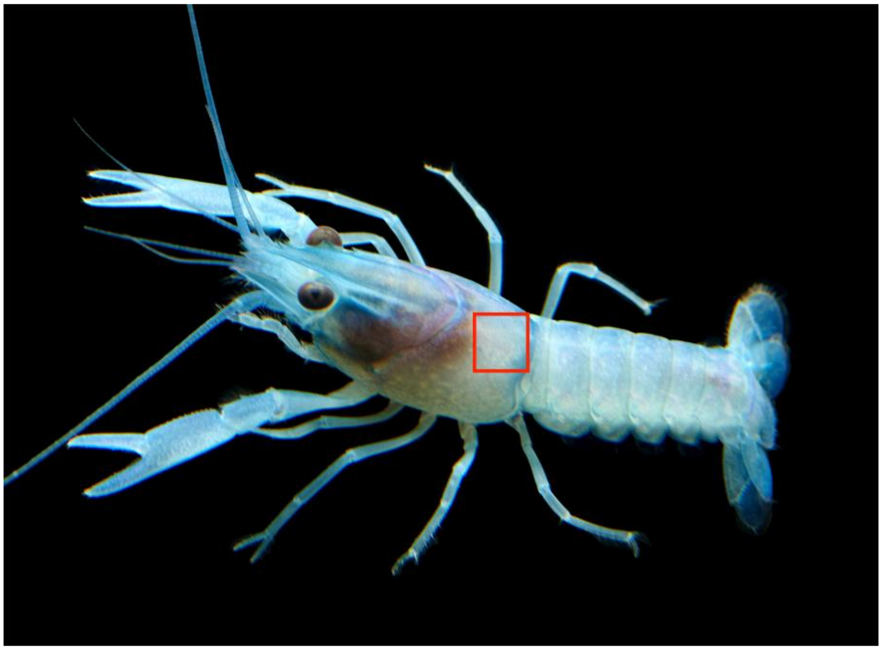

Supplement: Supplementary file 1 [file animals-14-00411-s001.zip › Supplementary_Material.pdf]
